# Fascia iliaca compartment block for postoperative pain after total hip arthroplasty: a systematic review and meta-analysis of randomized controlled trials

**DOI:** 10.1186/s12871-024-02476-y

**Published:** 2024-03-09

**Authors:** Mona Muhe Eldeen Eshag, Lina Omar Mahmoud Hasan, Salem Elshenawy, Mennatallah Samir Ahmed, Abd El-moneam Emad Mostafa, Yomna Ali Abdelghafar, Yusuf Jasim Althawadi, Najwa Medhat Ibraheem, Helmy Badr, Yossef Hassan AbdelQadir

**Affiliations:** 1 Faculty of Medicine, University of Bahri, Khartoum, Sudan; 2 Faculty of Dentistry, University of Jordan, Amman, Jordan; 3 Faculty of Medicine, Alexandria University, Alexandria, Egypt; 4 Faculty of Medicine, Benha University, Qalubia, Egypt; 5 Faculty of Medicine for girls, Alazhar University, Cairo, Egypt; 6 Faculty of Medicine, Tanta University, Tanta, Egypt

**Keywords:** Fascia iliaca compartment block, FICB, Total hip arthroplasty, Total hip replacement, Placebo, Postoperative pain, Opioid

## Abstract

**Background:**

Fascia iliaca compartment block (FICB) is one of the regional nerve blocks used to reduce pain after total hip arthroplasty (THA). We aim to assess the efficacy of FICB in reducing post-operative pain and opioid consumption.

**Methods:**

We searched PubMed, Web of Science, Cochrane Library, Embase, and Scopus on February 19, 2023, and we updated our search in august 2023 using relevant search strategy. Studies were extensively screened for eligibility by title and abstract screening, followed by full-text screening. We extracted the data from the included studies, and then pooled the data as mean difference (MD) or odds ratio (OR) with a 95% confidence interval (CI), using Review Manager Software (ver. 3.5).

**Results:**

FIBC significantly reduced analgesic consumption at 24 h (MD = -8.75, 95% CI [-9.62, -7.88] *P* < 0.00001), and at 48 h post-operatively. (MD = -15.51, 95% CI [-26.45, -4.57], *P* = 0.005), with a significant sensory block of the femoral nerve (*P* = 0.0004), obturator nerve (*P* = 0.0009), and lateral femoral cutaneous nerve (*P* = 0.002). However, FICB was not associated with a significant pain relief at 6, 24, and 48 h postoperatively, except at 12 h where it significantly reduced pain intensity (MD = -0.49, 95% CI [-0.85, -0.12], *P* = 0.008). FICB was also not effective in reducing post-operative nausea and vomiting (MD = 0.55, 95% CI [0.21, 1.45], *P* = 0.23), and was associated with high rates of quadriceps muscle weakness (OR = 9.09, % CI [3.70, 22.30], *P* = < 0.00001).

**Conclusions:**

FICB significantly reduces the total analgesic consumption up to 48 h; however, it is not effective in reducing post-operative pain, nausea and vomiting and it induced postoperative muscle weakness.

**Supplementary Information:**

The online version contains supplementary material available at 10.1186/s12871-024-02476-y.

## Introduction

Total hip arthroplasty (THA) has an established efficacy in treating pain and reconstructing joint function to improve the range of motion in patients with severe hip osteoarthritis [1]. It is a widely performed operation nowadays, with over 80,000 procedures performed in England, and 24,000 procedures in Canada annually [2, 3]. It is the treatment of choice for advanced pain of osteoarthritis after the failure of physical and medical therapy [1, 4]. In addition to osteoarthritis, it is also indicated in several disorders such as avascular necrosis, traumatic fracture in the neck of femur, congenital hip dislocation, and inflammatory arthroplasty [2]. 

One of the essences of modern hip reconstructive surgery is to achieve optimal pain control and restore the normal function of the affected joint [5]. The most common adverse effects after THA include postoperative pain, nausea and vomiting, and respiratory impairment, which may worsen the postoperative rehabilitation [6]. Delayed rehabilitation is associated with several complications, such as deep venous thrombosis, pulmonary embolism, and pulmonary infarction [7]. 

Pain control after THA is a debatable topic since there are no strong recommendations or uniform guidelines [8]. Uncontrolled pain is associated with delayed physical therapy, which prolongs patients’ rehabilitation period, increases the length and cost of hospital stay, and decreases patients’ satisfaction with the operation [9]. Opioids are established as the best treatment for post-operative pain; however, their serious side effects such as respiratory depression and the risk of dependence have increased the need to search for other safer alternatives [10]. 

Multimodal pain management approach is now considered the standard method to achieve optimal pain control, while minimizing the need for opioids and their adverse events [5]. It includes the use of oral pharmacological agents and different regional analgesic interventions [11]. The mainstay of oral analgesics is paracetamol and non-steroidal anti-inflammatory drugs, other studies suggest the use of gabapentin and anticonvulsants [5]. local infiltration anesthesia, femoral nerve block, epidural analgesia, and patients-controlled analgesia are the modern primary options for acute postoperative pain [5, 7, 12]. 

Fascia iliaca compartment block (FICB) is one of the regional nerve blocks used in THA [13]. It involves injecting the anesthetic agent under fascia iliaca to block femoral nerve and lateral femoral cutaneous nerve [14]. FICB is most commonly used for total knee arthroplasty procedure; however, the small number of published studies on its effect in reducing pain and opioid consumption after THA as well as the low quality of the present articles deems the FICB controversial [15]. Moreover, previous meta-analysis provided conflicting results on the efficacy of FICB [16–19]. So, the main aim of this study is to provide the most recent update on the efficacy of FICB after THA.

## Methods

This systematic review and meta-analysis was conducted according to the Preferred Reporting Items for Systematic Review and Meta-Analysis (PRISMA) guidelines and checklist [20]. We also followed the rules of the Cochrane Handbook for Systematic Reviews of Interventions [21].

### Literature search

We searched PubMed, Web Science, Cochrane Library, Embase, and Scopus on February 19, 2023 for published randomized controlled trials and we updated our search on august 28,2023. We used the following search strategy ((hip arthroplasty) OR (“Arthroplasty, Replacement, Hip“[Mesh]) OR (hip replacement) OR (Total Hip Replacement) OR (Hip Replacement Arthroplasties) OR (Hip Replacement Arthroplasty) OR (Hip Prosthesis Implantations) OR (THA) OR (total hip arthroplasty) OR (Total Hip Arthroplasties)) AND ((fascia iliaca block) OR (fascia iliaca) OR (fascia iliaca compartment block)). Finally, we manually screened the reference lists of the included studies for any eligible articles.

### Eligibility criteria and study selection

We included randomized controlled trials comparing FICB with placebo in patients undergoing total hip replacement. We excluded animal studies, cohort or case control reports, in vitro studies, overlapped datasets, conference abstracts, reviews, book chapters, theses, editorial letters and abstract only papers. After duplicate removal using Endnote software, two independent authors screened the title and abstract of the articles followed by full text screening of eligible articles. Conflicts were solved by consulting a third author.

### Data extraction

We extracted baseline demographic characteristics of the patients, summary of the main results of the included studies, and the following outcomes: pain intensity, total analgesic consumption, sensory block, and nausea and vomiting.

### Risk of bias assessment

Two authors independently assessed the quality of the included studies using Cochrane risk of bias 2 tool as described in Cochrane handbook, and the assessed domains included: Randomization process, Deviation from intended interventions, Missing outcome data, Measurement of the outcome, and Selection of the reported results [22]. 

### Data synthesis

We used Review Manager software version 5.4 for the meta-analysis, continuous outcomes were pooled using main difference (MD), dichotomous outcomes were pooled using odds ratio (OR), all with 95% confidence intervals (CIs). Heterogeneity between pooled studies was assessed using chi-square and I-square tests. The studies were considered heterogeneous at chi-square p-value < 0.1 and I^2^ > 50%. Fixed effect model was used for the analysis unless heterogeneity was detected in which case random effect model was used.

## Results

**Fig. 1:**
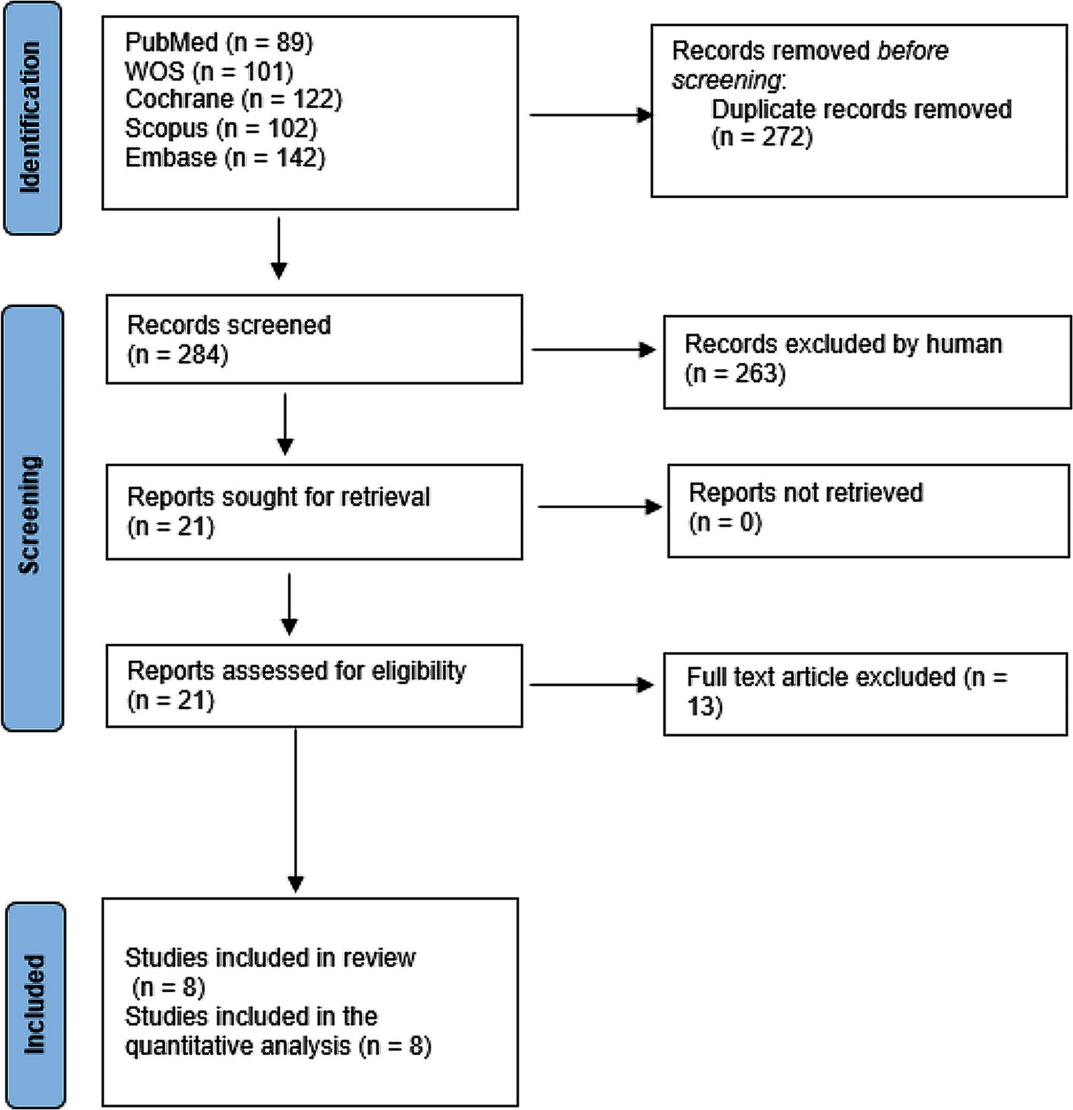
Shows the PRISMA flow chart

The literature search located 556 articles. We ran title and abstract screening then full text screening that resulted in eight articles finally included in our meta-analysis (See PRISMA flow diagram; Fig. 1). [23–29, 37]

Baseline characteristics of the included studies are shown in Table 1. While summery and main results are shown in Table 2.

**Table 1:** Shows baseline characteristics of the patients in the included studies

Study ID	Study group	Age(year) Mean(SD)	BMI (kg/m2) Mean(SD)	Sex, NO. of females (%)	Physical status		
					ASAI N(%)	ASAII N(%)	ASAIII N(%)
Bober2020 [27]	FICB	62.9(13.03)	–	31(53%)	–	–	–
Chen 2023 [37]	FICB	74(6.13)	22.64(2.60)	29(64.4%)	0 (0%)	31 (68.9%)	14(31.1%)
	Placebo	72(7.7)	22.12(2.09)	26(59.1%)	0 (0%)	31 (70.5%)	13(29.5%)
	Placebo	63.9(13.03)	–	36(60%)	–	–	–
Deniz2014 [23]	FICB	59.1 ± 13.1		12(60%)	12(60%)	6(30%)	2(10%)
	Placebo	62.2 ± 13.7		12(60%)	5(25%)	10(50%)	5(25%)
Desmet2017 [26]	FICB	60.4(10.08)	27.3(4)	23(54.76%)	–	–	–
	Placebo	66.5(12.4)	27.3(4.5)	29(67.44%)	–	–	–
Gola2021 [24]	FICB	65(12)	27(3)	29(58%)	–	23(46%)	27(54%)
	Placebo	65(9)	28.1(2.9)	28(56%)	–	34(68%)	16(32%)
Liu2020 [28]	FICB	70.05(5.52)	23.24(3.13)	11(29.73%)	–	–	–
	Placebo	70(5.69)	23.05(2.89)	12(32.43%)	–	–	–
Shariat2013 [29]	FICB	61(9)	30(7)	9(56.25%)	–	–	–
	Placebo	57(13)	30(7)	8(50%)	–	–	–
Stevens2007 [25]	FICB	68.7(9.7)	–	7(32%)	3(14%)	11(52%)	7(33%)
	Placebo	66.8(9.1)	–	11(50%)	5(23%)	12(55%)	5(23%)

BMI: body mass index, SD: standard deviation, ASA: American society of Anaesthesiology, Data are presented as mean and (SD) or number and (%)

**Table 2:** Shows a summary of the included studies and their main findings

Study ID	Sample size	Study location	Hip arthroplasty setting	Type of FICB	Type of anesthesia	Intervention	Control	Study design	Duration of follow up	Main results
Bober2020 [27]	119	USA	Elective	Ultrasound guided	Epidural anesthesia	40 ml of 0.25% bupivacaine	Placebo	RCT	4 weeks	No significant difference in the morphine equivalents consumed and average pain scores during the first 24 h.
Chen 2023 [37]	89	China	Elective	Ultrasound guided	Spinal anesthesia	40 mL 0.5% ropivacaine	Placebo	RCT	48 h	FICB significantly increased the duration of analgesia and improved 24-h QoR, but reduced postoperative muscle strength.
Deniz2014 [23]	70	Turkey	Elective	Ultrasound guided	General anesthesia	30 ml of 0.25% bupivacaine	Placebo	RCT	24 h	FICB significantly reduces analgesic consumption, but no significant difference in VAS scores after the first two hours.
Desmet2017 [26]	85	Belgium	Elective	Ultrasound guided	General anesthesia	40 mL of 0.5% ropivacaine	Placebo	RCT	48 h	FICB Significantly reduces morphine consumption at 24 and 48 h, and pain scores at 1, 2, 4, and 24 h postoperatively.
Gola2021 [24]	100	Poland	Elective	Ultrasound guided	Spinal anesthesia	40 mL of 0.375% ropivacaine solution with adrenaline at a dose of 5 µg/mL of solution.	Placebo	RCT	48 h	FICB significantly reduce analgesic consumption, and pain scores at all-time points except for 48 h.
Liu2020 [28]	119	China	Not determined	Ultrasound guided	General anesthesia	30 ml of 0.2% ropivacaine	Placebo	RCT	72 h	The pain scores at all-time points were significantly lower in the FICB group
Shariat2013 [29]	32	USA	Elective	Ultrasound guided	General anesthesia	30 mL of 0.5% ropivacaine	Placebo	RCT	24 h	Pain scores were significantly lower in FICB at 24 h. No significant difference in morphine consumption between the two groups
Stevens2007 [25]	44	Australia	Elective	Two pop method	Spinal anesthesia	30 ml of 0.5% bupivacaine with 1:200,000 adrenaline, 150 µg clonidine and 9 ml of 0.9% saline (total volume 40 ml).	Placebo	RCT	24 h	No significant difference in pain scores between the two groups. At both the 12 and 24-hour interval there was a significant decrease in morphine use by the FICB group.

The overall quality of the included studies was moderate. Authors’ judgment of risk of bias assessment domains is shown in Table 3.

**Table 3:** Risk of bias assessment

Study ID	Domain 1. Randomization process	Domain 2. Deviations from intended interventions 1	Domain 2. Deviations from intended interventions 2	Domain 3. Missing outcome data	Domain 4. Measurement of the outcome	Domain 5. Selection of the reported result	Domain 6. Overall Bias
Bober2020 [27]	Low risk	Low risk	Low risk	Low risk	Low risk	Low risk	Low risk
Chen 2023 [37]	Low risk	Low risk	Low risk	Low risk	Low risk	Low risk	Low risk
Deniz2014 [23]	Some concerns	Low risk	Low risk	Low risk	Some concerns	Low risk	Some Concerns
Desmet2017 [26]	Low risk	Low risk	Low risk	Low risk	Low risk	Low risk	Low risk
Gola 2021 [24]	Some concerns	Low risk	Low risk	Low risk	Some concerns	Low risk	Some Concerns
Liu 2020 [28]	Some concerns	Some concerns	Low risk	High risk	Some concerns	Some concerns	High risk
Shariat2013 [29]	Low risk	Low risk	Low risk	Low risk	Low risk	Low risk	Low risk
Stevens2007 [25]	Low risk	Some concerns	Low risk	High risk	Low risk	Low risk	High risk

### Pain intensity

#### 6 H post-operatively

**Fig. 2:**
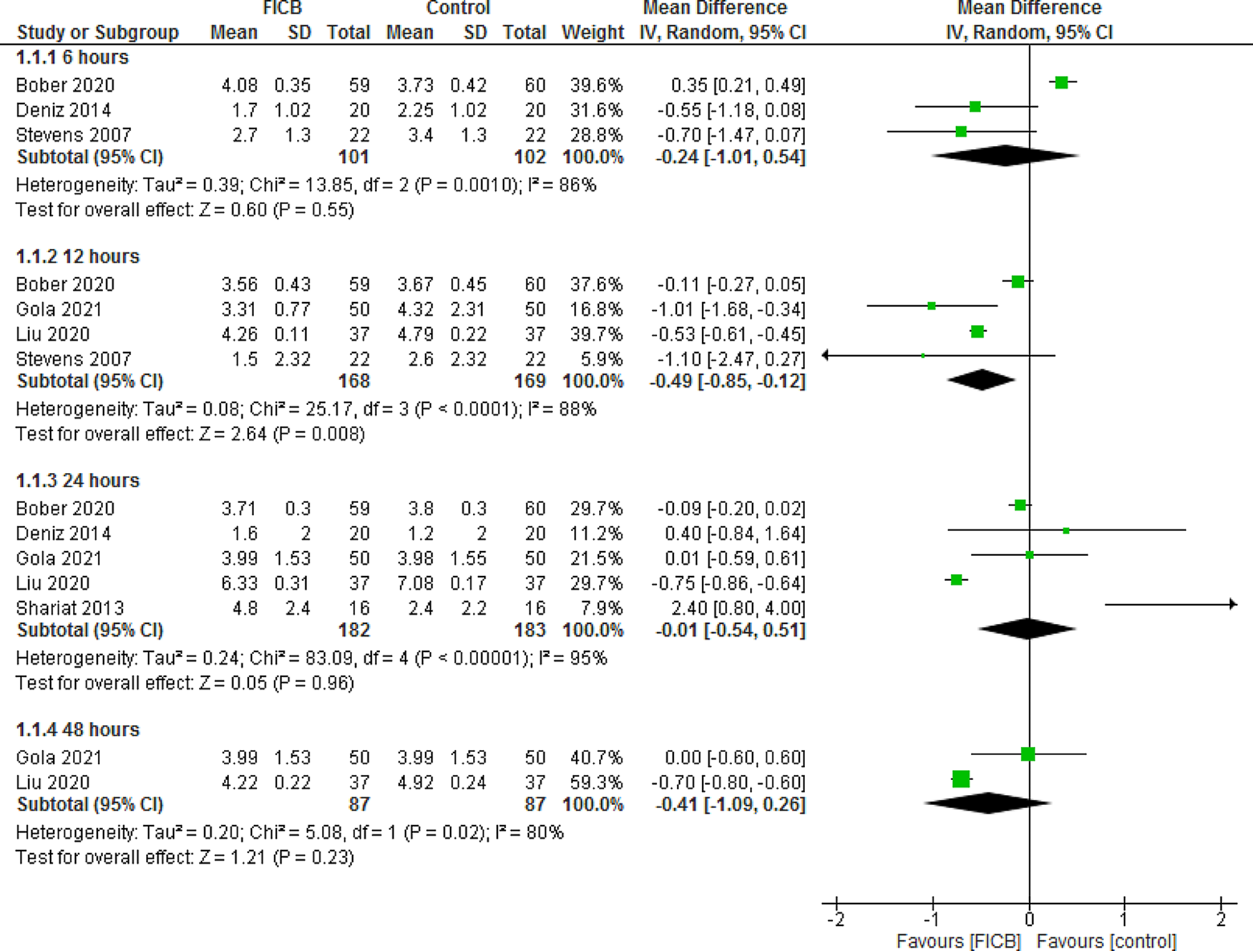
Shows of the forest plot of pain scores at 6, 12, 24, and 48 h post-operatively

The pooled results showed no statistically significant difference between FICB and placebo regarding pain scores at 6 h post-operatively (MD = -0.24, 95% CI [-1.01, 0.54], *P* = 0.55), however the pooled data were heterogeneous (*P* = 0.0010, I² = 86%), and the heterogeneity was solved by excluding Bober 2020 (*P* = 0.77, I² = 0%), and the effect estimate became significant (MD = -0.61, 95% CI [-1.10, -0.12], *P* = 0.01). (Fig. 2) [27].

#### 12 H post-operatively

FICB was significantly better than placebo in reducing pain intensity at 12 h post-operatively (MD = -0.49, 95% CI [-0.85, -0.12], *P* = 0.008), the pooled data were heterogeneous (*P* < 0.0001, I² = 88%), and the heterogeneity was resolved after excluding Bober 2020 (*P* = 0.28, I² = 22%) and the effect estimate remained significant (MD = -0.63, 95% CI [-0.93, -0.33], *P* < 0.0001). (Fig. 2) [27].

#### 24 H post-operatively

No significant difference between FICB and placebo was detected at 24 h post-operatively (MD = -0.01, 95% CI [-0.54, 0.51], *P* = 0.96), the pooled data were heterogeneous (*P* < 0.00001, I² = 95%), and the heterogeneity could not be solved by “leave-one-out” method. (Fig. 2)

#### 48 H post-operatively

No significant difference between FICB and placebo was detected at 48 h post-operatively (MD = -0.41, 95% CI [-1.09, 0.26], *P* = 0.23), the pooled results were heterogeneous (*P* = 0.02, I² = 80%), and the heterogeneity could not be solved as it includes only two studies. (Fig. 2)

### Analgesic consumption

#### 24 H post-operatively

**Fig. 3:**
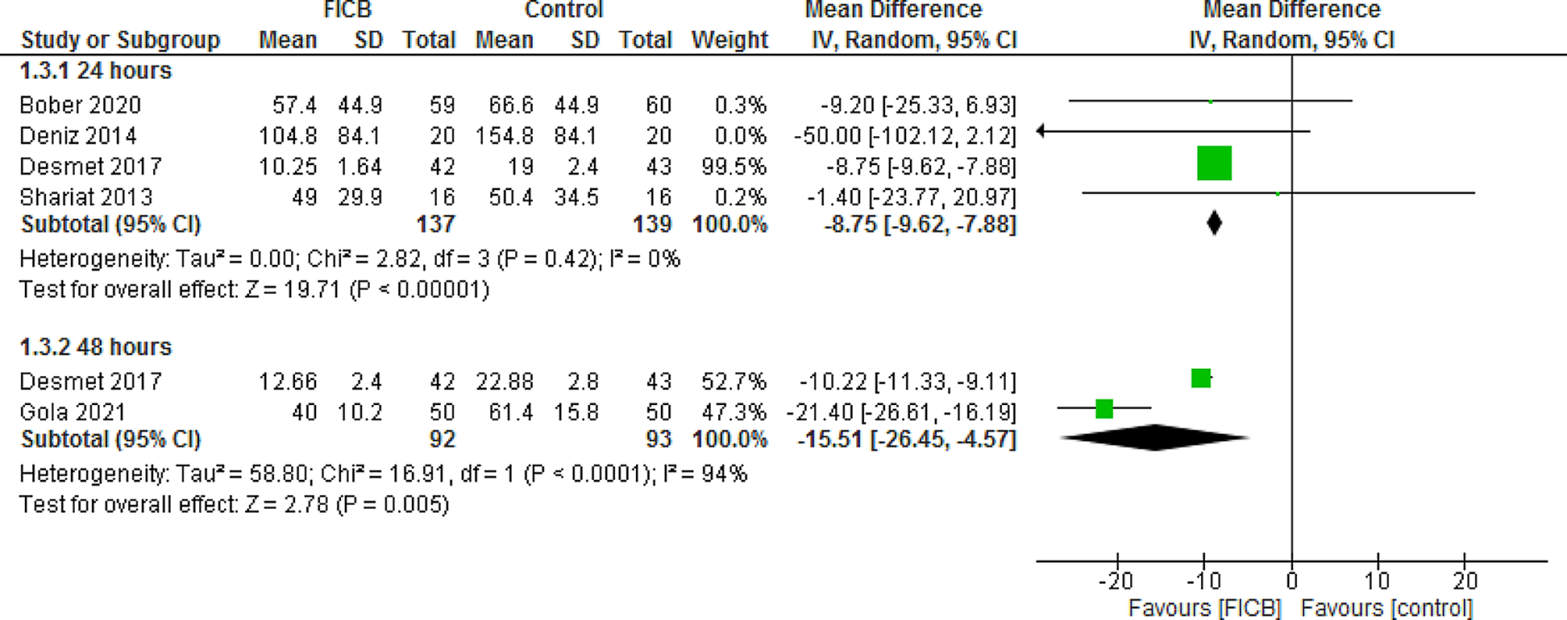
Shows of the forest plot of analgesic consumption after 24 and 48 h post-operatively

The pooled results showed statistically significant reduction in analgesic consumption with FICB at 24 h post-operatively (MD = -8.75, 95% CI [-9.62, -7.88] *P* < 0.00001), and the pooled data were homogenous (*P* = 0.42, I² = 0%). (Fig. 3).

#### 48 H post-operatively

FICB significantly reduced analgesic consumption at 48 h post-operatively (MD = -15.51, 95% CI [-26.45, -4.57], *P* = 0.005), the pooled data were heterogeneous (*P* < 0.0001, I² = 94%), and the heterogeneity couldn’t be solved as there are only two studies. (Fig. 3).

### Sensory block

**Fig. 4:**
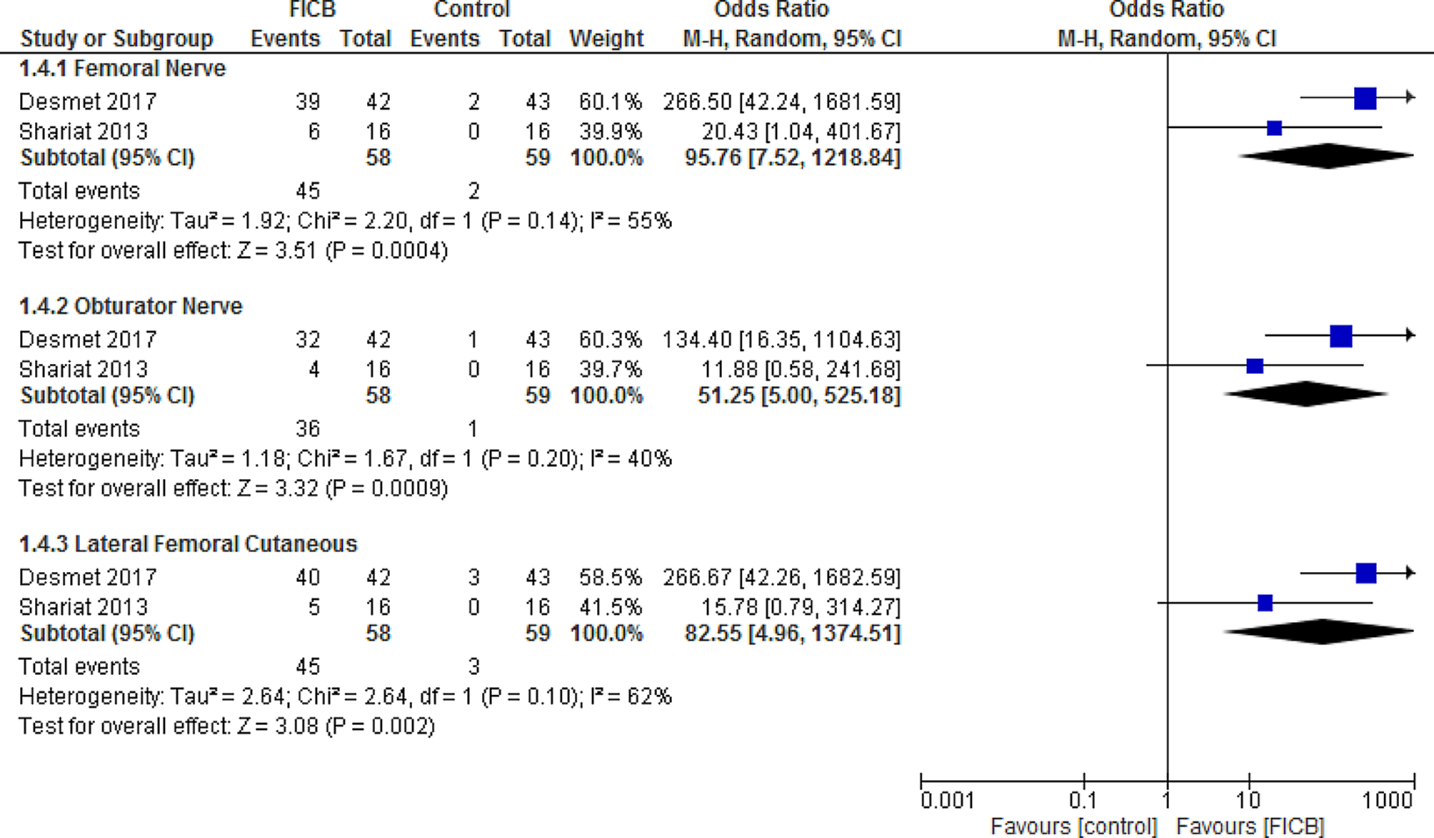
Shows of the forest plot of sensory block of the femoral nerve, the obturator nerve, and the lateral femoral cutaneous nerve

The pooled odds ratio showed that sensory block was significantly more frequent in FICB group than placebo group in all three nerves, Femoral nerve (OR = 95.76, 95% CI [7.52, 1218.84], *P* = 0.0004), heterogeneous data (*P* = 0.14, I² = 55%), Obturator nerve (OR = 51.25, 95% CI [5.00, 525.18], *P* = 0.0009), homogenous data (*P* = 0.20, I² = 40%), and Lateral femoral cutaneous (OR = 82.55, 95% CI [4.96, 1374.51], *P* = 0.002), heterogeneous data (*P* = 0.10, I² = 62%), and the heterogeneity couldn’t be solved as there are only two studies. (Fig. 4)

### Nausea and vomiting

**Fig. 5:**
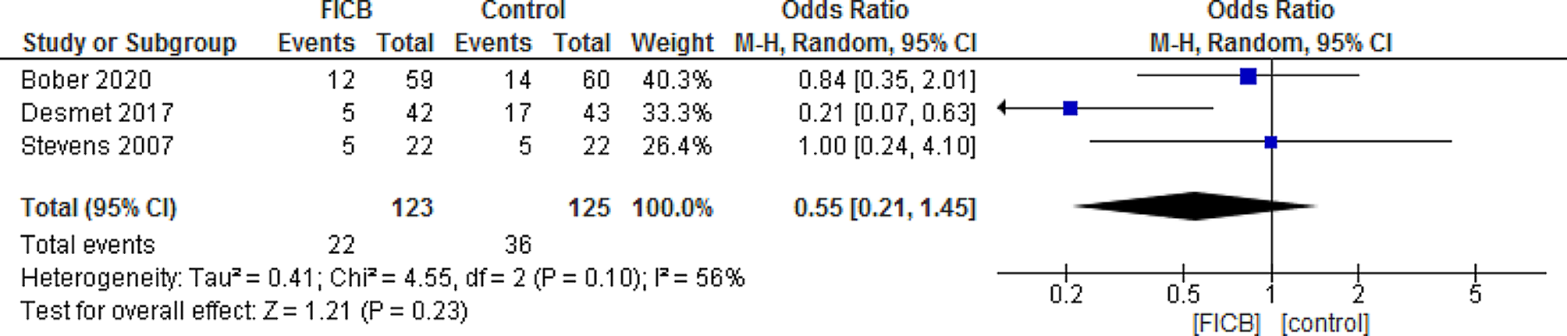
Shows the effect of FICB on the post-operative nausea and vomiting

The pooled odds ratio showed that nausea and vomiting were not significantly different between FICB group and placebo group (OR = 0.55, 95% CI [0.21, 1.45], *P* = 0.23), however the pooled data were heterogeneous (*P* = 0.10, I² = 56%), and the heterogeneity was resolved by excluding Desmet 2017 (*P* = 0.84, I² = 0%), and the effect estimate remained not significant (OR = 0.88, 95% CI [0.42, 1.85], *P* = 0.74). (Fig. 5) [26]. 

### Muscle weakness

**Fig. 6:**
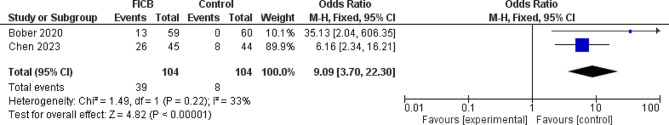
Shows the postoperative quadriceps muscle weakness

The pooled odds ratio showed that FICB was associated with significantly higher rate of quadriceps muscle weakness than the control group (OR = 9.09, % CI [3.70, 22.30], *P* = < 0.00001). the pooled data were homogeneous (*P* = 0.22, I² = 33%). (Fig. 6)

## Discussion

On assessing the efficacy of fascia iliaca compartment block (FICB) in total hip arthroplasty (THA), we found that FICB significantly reduced the total analgesic consumption after 24 and 48 h postoperatively compared to placebo with a more significant sensory block in the femoral, obturator and lateral femoral nerves. However, we found no significant difference in the pain intensity between the FICB and the placebo group at 6, 24, and 28 h postoperatively, except at 12 h postoperatively where FICB significantly reduced pain intensity. FICB was also not effective in post-operative nausea and vomiting. Finally, FICB was associated with the major side effect, quadriceps muscle weakness.

On analyzing our included studies, all of them were randomized control trials (RCT), compared between FICB and placebo. They used different methods for anesthesia during the surgery as follows: Stevens et al. and Gola et al. used spinal anesthesia [24, 25], Bober et al. used epidural anesthesia [27]. , and the other four studies used the standard general anesthesia [26, 28, 29]. As the regards the analgesic technique used in our studies, four studies used the standard infra-inguinal approach for FICB and showed conflicting results [27–29]. Sheriat et al. and Bober et al. showed no significant effect in pain reduction nor analgesic consumption in the FICB group [27, 29]; however, liu et al. and Deniz et al. showed a significant effect in the FICB group [28]. The other three studies used the modified supra-inguinal approach, and all of them showed a significant reduction in the pain intensity and the analgesic consumption in the FICB group [24–26]. 

The supra-inguinal approach is gaining more popularity in the clinical practice lately. In a recent study by Kumar et al., they directly compared the infra-inguinal to the supra-inguinal approach after THA, and concluded that the supra-inguinal approach was associated with a significant reduction in the 24 hours’ post-operative morphine consumption (6.95 ± 2.14 vs. 10.50 ± 2.24, p = < 0.001 respectively) [30]. This efficacy is mainly attributed to the higher rates of target nerves block in the supra-inguinal method due to more proximity to the lumbar plexus, which was also observed in the study by lee et al. [31] Moreover, a longitudinal approach instead of the conventional transverse approach is believed to show a more cranial spread of the local anesthesia with higher rates of obturator nerve block [32]. 

FICB is an analgesic technique that targets the nerves of the anterior and the lateral compartments of the thigh. On the other hand, THA is a surgical procedure that has many surgical approaches; hence, the type of the surgical approach may affect the analgesic outcome of the FICB postoperatively. Recent studies show that the direct anterior approach was associated with a faster post-operative rehabilitation, shorter hospital stay and a better pain relief [33, 34]. All our included studies applied the anterior approach for THA except Bober et al. and Gola et al. [24, 27] Bober at al. investigated the effect of FICB after direct posterior approach of THA, and found it ineffective in reducing postoperative pain score and opioid consumption [27]. However, Gola et al. applied a modified posterolateral approach, in which FICB showed a great efficacy in reducing post-operative pain and opioid consumption, with a significantly lower hospital stay and a higher level of patients’ satisfaction [24]. We did a sensitivity analysis on the posterior approach done by Bober et al. as it caused significant heterogeneity in many of our outcomes, and results of the analgesic consumption became insignificant after 48 h post-operatively as shown in appendix 1.

All our studies used a similar dose of ropivacaine or bupivacaine ranging from 0.2 to 0.5% in 30 or 40 ml. However; in the study by Desmet et al., they used a higher dose reaching a mean of 2.6 mg/kg ropivacaine (range, 2–3.4 mg/kg) [26]. Their results showed a great reduction in morphine consumption of 46% after 24 h and of 45% after 48 h. We performed a sensitivity analysis on the effect of high dose analgesia and the effect of FICB in reducing the total analgesic consumption became insignificant after 24 h as shown in appendix 1 [26]. On the other hand, Liu et al. compared the combined effect of pre-operative dexmedetomidine (DEX), an α2-adrenergic receptor agonist that has analgesic properties, and post-operative FICB to the use of post-operative FICB alone, and found that the use of the combination was significantly better in reducing the pain scores up to 72 h post-operatively [28]. It also reduced the total opioid consumption post-operatively, improved sleep quality, and reduced the serum level of inflammatory markers, which makes this combination a possible alternative in local analgesia [28]. Another study by Deniz et al. compared the use of FICB to another technique (3 in 1 block), in which the local analgesia is injected directly into the inguinal ligament, and it was inferior to FICB in reducing post-operative opioid consumption [23]. 

FICB showed a tendency towards decreasing post-operative nausea and vomiting, however, our results were statistically not significant; although we couldn’t include Gola et al. and Liu et al. in the analysis as they reported nausea and vomiting separately, and the data of Liu et al. wasn’t applicable for pooling in our analysis [24, 28]. However, Gola et al. found a significant decrease in nausea and vomiting in the FICB group, consistent with the results of Desmet et al. [24, 26] On the other hand, a major adverse event, quadriceps weakness, was reported in two studies. It is a relatively rare adverse events due to femoral nerve injury. Wang et al. reported the same complication after applying an ultra-sound guided supra-inguinal FICB [35, 36]. The data remain unclear about this complication and further data are needed to know the exact predisposing factors for it to be avoided in future research.

Our results agree with previous meta-analyses by Zhang et al., Goa et al., and Cai et al. in the proved efficacy of FICB in reducing post-operative opioid consumption at 24 and 48 h [16–18]. However, their results on pain scores post-operatively are conflicting with ours. Our results agree with Goa et al. and Cai et al. in the reduction of pain scores at 12 h and wearing of this effect at 24 h [17, 18]. On the other hand, Zhang et al. stated that the first eight hours showed the peak effect of pain reduction in the FICB group and no significant pain reduction was detected afterwards, which disputes with our results at six hours that showed no significant reduction [16]. All three studies showed a significant decrease in nausea and vomiting in the FICB group [16–18]. We couldn’t prove this effect, although there was a tendency to a more significant decrease in the FICB group. Zhang et al. and Goa et al. showed a significant reduction in the hospital stay length; however, data were not sufficient in our study to perform this analysis [16, 18]. However, we have major methodological differences from them. First, they included conference abstracts, observational studies and non-English articles, while we only included RCTs only available in an English text. Second, they included a paper investigating the effect of FICB on hemi arthroplasty besides total arthroplasty, which may cause heterogeneity in the analysis with unreliable results.

Another recent meta-analysis by Dai et al. compared FICB and placebo in addition to other post-operative analgesic methods [19]. The total analysis retrieved no statistically significant effect of FICB over placebo or any other analgesic methods [19]. Similarly, in their subgroup analysis on the effect of FICB compared to placebo, they didn’t find any significant effect in pain reduction at 24 h, analgesic consumption at 24 h, or complication rate [19]. However, we disagree with their results, as we included more studies in our analysis and FICB proved to be effective in reducing post-operative analgesic consumption after 24 and 48 h. We also compared the effect at longer duration (48 h), which was significant in the reduction of opioid consumption, but not significant in the pain relief. Moreover, they analyzed the complication rate as a single outcome without specifying the type of the complication, which may be misleading.

Our study has some major strength points. First, we present the most recent update on the efficacy of FICB in pain control after THA, which contradicts with the previous meta-analysis and thus affects the future clinical decision. Second, we included only RCTs, which represent a high level of evidence. Third, we used the most recent Cochrane risk of bias tool (ROB2), and quality of the included studies were overall moderate, we perform a sensitivity analysis on the high quality studies, the data are provided in appendix 2. However, our study was limited by the heterogeneity regarding the type of the anesthesia during the operation, the approach of the THA surgery, the type of the technique, the total volume and dose used during anesthesia. However, we did a sensitivity analysis on the surgical approach and the dose of the local analgesia to solve this heterogeneity, and provided the data in appendix 1. Finally, we couldn’t perform a subgroup analysis on the type of anesthesia during the operation and the type of the technique, due to the limited number of studies in each group.

To conclude, FICB is an effective analgesic method in reducing post-operative analgesic consumption after THA, by effectively blocking all the sensory supply to the femoral, obturator and lateral femoral nerves. However, different factors may interfere with this effect including the approach of the operation and the type of technique. Thus, more clinical trials are needed to figure out the optimal application for this analgesic procedure.

### Electronic supplementary material

Below is the link to the electronic supplementary material.

Supplementary Material 1

Supplementary Material 2

## Data Availability

The datasets used and/or analyzed during the current study are available from the corresponding author upon reasonable request.
