# Supplementary material for: Fascia iliaca compartment block for postoperative pain after total hip arthroplasty: a systematic review and meta-analysis of randomized controlled trials
Source: BMC Anesthesiol. 2024 Mar 9;24:95. doi: 10.1186/s12871-024-02476-y (PMC10924383; doi:10.1186/s12871-024-02476-y)

Figure 1: Sensitivity analysis of high quality studies on post-operative pain intensity.


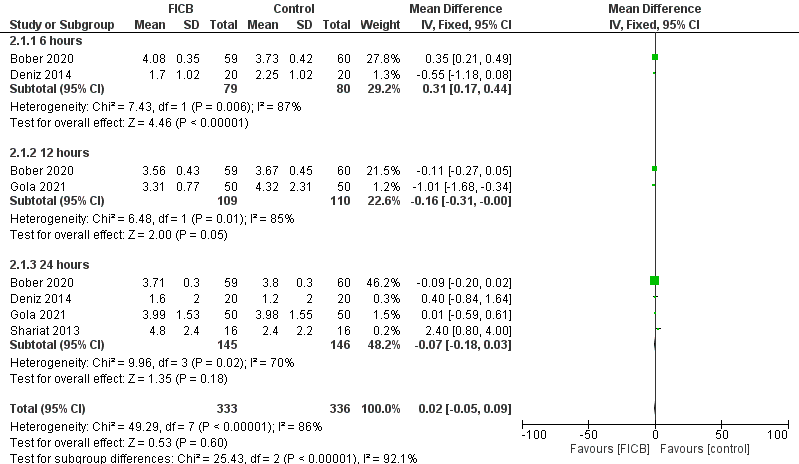


Figure 2: Sensitivity analysis of high quality studies on postoperative nausea and vomiting.


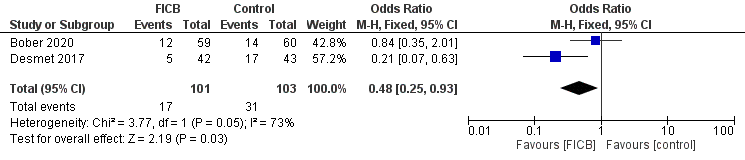

Supplement: Supplementary file 2 — Supplementary Material 2 [file 12871_2024_2476_MOESM2_ESM.docx]
